# One-Pot Synthesis of Ag/Quaternary Ammonium Salt Co-Decorated Mesoporous Silica Nanoparticles for Synergistic Treatment of Cancer and Bacterial Infections

**DOI:** 10.3389/fbioe.2022.875317

**Published:** 2022-07-19

**Authors:** Hanyuan Zhang, Jianxiang Xu, Xu Zhang, Teng Wang, Dairan Zhou, Wei Shu, Tingting Zhao, Weijun Fang

**Affiliations:** ^1^ Department of Orthopedics, Department of Sports Medicine and Arthroscopic Surgery, The First Affiliated Hospital of Anhui Medical University, Hefei, China; ^2^ School of Basic Medical Sciences, Anhui Medical University, Hefei, China

**Keywords:** synergistic treatment, anticancer, antibacterial activity, mesoporous silica nanoparticles, quaternary ammonium salts, Ag

## Abstract

Developing drug delivery nanosystems with both anticancer and antibacterial effects is of great clinical value. Herein, we report a facile approach to synthesize Ag and quaternary ammonium salt (QAS) co-decorated mesoporous silica nanoparticles (MSNs), namely, Ag/QAS-MSNs, for synergistic treatment of cancer and bacterial infections. *In vitro* studies demonstrated that Ag/QAS-MSNs not only had a strong antibacterial activity against the bacterial pathogens but also could efficiently induce cancer cell death through an apoptotic pathway. Moreover, *in vivo* combination therapy with Ag and QAS in Ag/QAS-MSNs was also tested in a nude mouse tumor model, and a significant synergistic anticancer effect, which is superior to that obtained by therapy with Ag-MSNs or QAS-MSNs alone, was achieved. Such excellent anticancer and antibacterial activity of Ag/QAS-MSNs could be attributed to the synergistic effect of Ag ions and QAS. Thus, Ag/QAS-MSNs have a promising future as potent anticancer agents with high antibacterial performance.

## 1 Introduction

Cancer is a life-threatening disease with a great worldwide impact. According to statistics, approximately 19.29 million new cancer cases and 9.96 million cancer deaths have occurred in 2020 worldwide (Siegel et al., 2020). At present, chemotherapy, surgery, and radiotherapy are still the three dominant treatment approaches. However, owing to drug resistance and lacking targeted ability, most cancer patients often have a poor prognosis. Moreover, numerous studies have shown that cancer-associated bacteria can seriously reduce the efficiency of cancer treatments (Wallace et al., 2010; Deweerdt, 2015; Liang et al., 2017; Yougbare et al., 2019). For instance, some bacteria in the tumor microenvironment can metabolize chemotherapeutic drugs or regulate the autophagy of tumor cells, resulting in enhanced tumor cells becoming resistant to chemo-drugs (Geller et al., 2017; Yu et al., 2017). Meanwhile, cancer patients with a weakened immune system are more susceptible to bacterial infections (Bullman et al., 2017; Harper, 2019). To address these challenges, the development of a new strategy based on a single element for both treating cancer and killing bacteria is of great clinical significance.

As is well known, quaternary ammonium salts (QASs) are composed of a hydrophilic ammonium cation head and a hydrophobic alkyl tail. Based on this unique structure, QASs can adhere to the negatively charged cellular membrane of microorganisms by electrostatic interaction, and then the hydrophobic alkyl tail can incorporate into the membrane lipid bilayer, resulting in cell membrane damage or cell membrane protein denaturation. Hence, QASs are bactericidal and fungicidal, as well as active toward tumor cells (He et al., 2010; Banerjee et al., 2011; He et al., 2011; Oblak et al., 2019; Kwasniewska et al., 2020; Tang et al., 2020; He et al., 2021). To date, several groups have demonstrated that the QAS could be used as a potential cancer-specific compound against head and neck cancer cell lines (Ito et al., 2009; Meena et al., 2017). In addition to, several nanoparticle-based systems also exhibited both anticancer and antibacterial abilities, such as iron oxide nanoparticles and carbon-related materials (Chu et al., 2013; Huang et al., 2015; Yu and Chi, 2020; Bayda et al., 2021; Te and Wei, 2022). In contrast, silver (Ag) nanoparticles possess a remarkable antibacterial and anticancer activity and have been widely used in medicine with significant applications including antimicrobial, bioimaging, and cancer therapy (Tang and Zheng, 2018; Deshmukh et al., 2019; Ahn and Park, 2020; Eid et al., 2020; Xu et al., 2020; Hsu et al., 2021; Bi et al., 2022). Compared to conventional anticancer drugs, QASs and Ag nanoparticles are cheaper and easier to use. These advantages make QASs and Ag nanoparticles as promising cancer therapeutic candidates.

A way to improve the efficacy of anticancer treatment is by the dual loading of drugs on a single nanovehicle, exploiting the synergetic effect of drugs (Oliveira et al., 2016; Kang et al., 2018; Montalvo-Quiros et al., 2019; Chen et al., 2020; Lima-Sousa et al., 2020; Song et al., 2020; Tan et al., 2020; Shen et al., 2021; Galhano et al., 2022). However, developing a dual drug-loaded nanosystem still remains a significant challenge. Herein, we designed a facile one-pot route to fabricate Ag and QAS co-decorated mesoporous silica nanoparticles (MSNs) (Ag/QAS-MSNs) for simultaneous fighting cancer and bacterial infections. The synthesized nanoparticles were characterized to study their physio-chemical properties using transmission electron microscopy (TEM), scanning electron microscopy (SEM), X-ray diffraction (XRD), energy dispersive spectroscopy, Fourier transform infrared spectroscopy (FTIR), and thermogravimetric analysis (TGA). We also thoroughly tested their anticancer *in vitro* and antibacterial activity against pathogenic Gram-negative (*Escherichia coli* JM109) and Gram-positive strains (*Staphylococcus aureus*). Moreover, the therapeutic effect and biosafety of Ag/QAS-MSNs were further studied *in vivo*.

## 2 Experimental Section

### 2.1 Materials and Methods

Tetraethylorthosilicate (TEOS, Alfa Aesar, United States), N-(aminoethyl)-amino-propyl trimethoxysilane (TSD, Alfa Aesar, United States), silver nitrate (AgNO_3_, Aladdin, China), hexadecyltrimethylammonium hydroxide salt (CTA-OH, 10% in water, Aladdin, China), formalin (HCHO, 40%, Sinopharm, China) ammonium nitrate (NH_4_NO_3_, Sinopharm, China), ammonium aqueous solution (NH_3_·H_2_O, 25%,Sinopharm, China), methanol (CH_3_OH, Sinopharm, China), and ethanol (C_2_H_5_OH, Sinopharm, China) were used as received without further purification. The bacterial strains (*Escherichia coli* JM109 and *Staphylococcus aureus*) were obtained from the Department of Microbiology, Anhui Medical University (China).

### 2.2 Instruments and Characteristics

SEM images and EDX spectra were captured on a Hitachi S4800 scanning electron microscope. TEM images were captured on a TECNAI F-30 high-resolution transmission electron microscope. XRD data were obtained in a PANalytical X’Pert diffractometer with Cu-Kα radiation from 10° to 80°. The concentrations of silver ions were tested using a inductively coupled plasma–atomic emission spectrophotometer (ICP-AES, IRIS Intrepid II XSP). The surface properties of the samples were determined using FTIR spectrum (Perkin-Elmer, Spectrum spectrometer), zeta potential (Nano ZS & MPT-2, Malvern), surface area, and pore size (Micromeritics Instrument Corp. ASAP 2020). Fluorescence micrographs were obtained on an Olympus IX71 fluorescence microscope.

### 2.3 Preparation of Ag/QAS Co-Decorated Mesoporous Silica Nanoparticles (Ag/QAS-MSNs)

**FIGURE 1:**
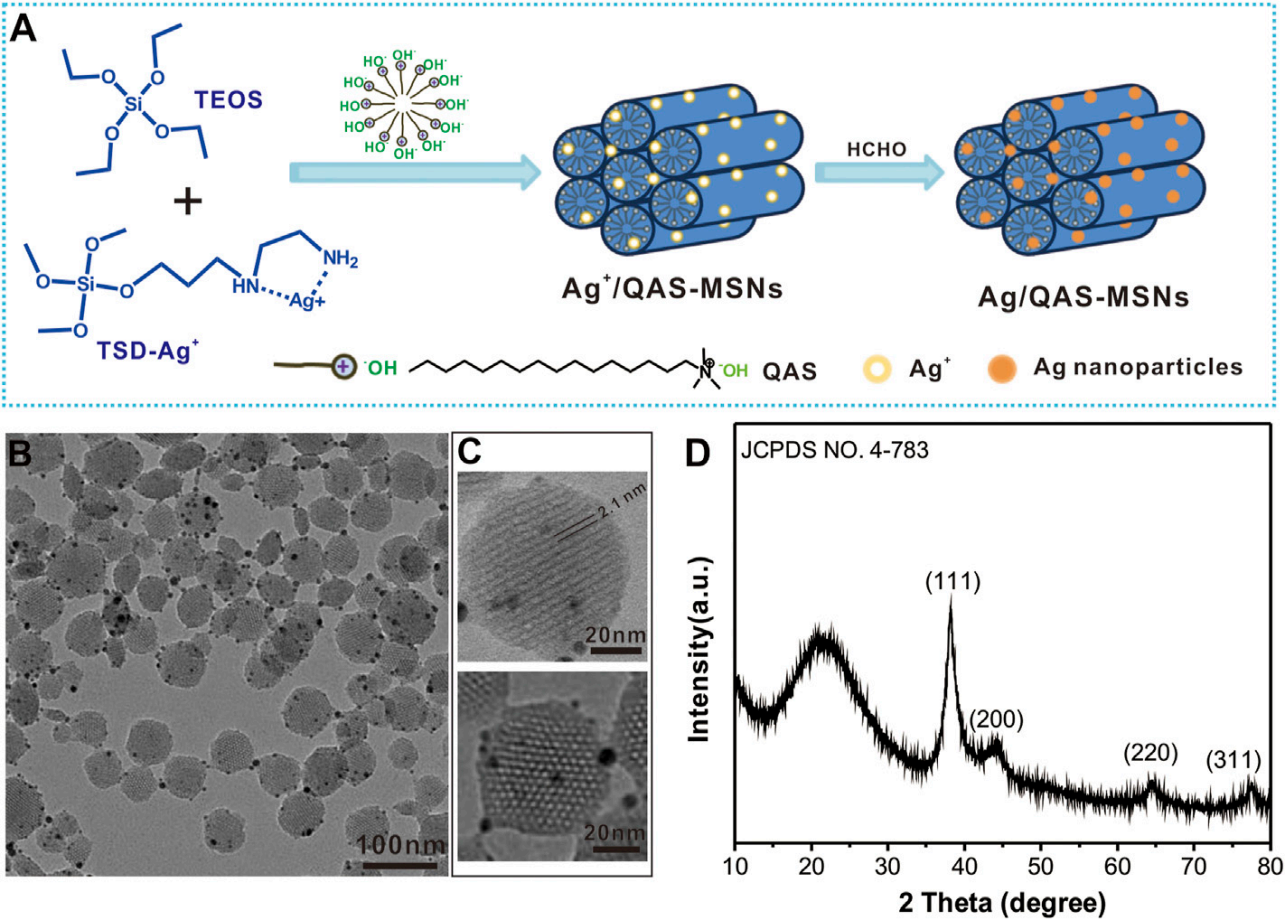
**(A)** Schematic illustration for the synthesis of Ag/QAS co-decorated MSNs. **(B)** TEM image of Ag/QAS-MSNs. **(C)** HRTEM images of Ag/QAS-MSNs. **(D)** XRD patterns of Ag/QAS-MSNs.

The MSNs co-decorated with Ag nanoparticles and hexadecyltrimethylammonium hydroxide salts were prepared via a one-pot method (Tian et al., 2014) (Figure 1A). A solution of CTA-OH (10% in water, 3.0 ml) and water (80.0 ml) was preheated to 80°C. Then, 0.833 ml of TEOS in ethanol (5.0 ml) and 0.25 ml of TSD and 30 mg of AgNO_3_ in water (2.0 ml) were simultaneously added into the above solution with stirring for 2 h. Further, 2 ml of formalin was added into the mixture with stirring for an additional 1 h at 80°C to reduce the loaded Ag ions into Ag nanoparticles. The final products were washed by ethanol and water for several times and dried in an oven at 50°C overnight.

### 2.4 Removal of Quaternary Ammonium Salts From Ag/QAS Co-Decorated Mesoporous Silica Nanoparticles

Ag/QAS-MSNs were dispersed in 10 ml of NH_4_NO_3_ ethanol solution (20 mg/ml) (Fang et al., 2012). The mixture was heated to 45°C under stirring for 4 h, and then the products (Ag-MSNs) were collected by centrifugation and washed with ethanol several times. The procedures were repeated three times.

### 2.5 Removal of Ag Nanocrystals From Ag/Quaternary Ammonium Salts Co-Decorated Mesoporous Silica Nanoparticles

The removal of the Ag nanocrystals from Ag/QAS-MSNs was performed by adding Ag/QAS-MSNs in a solution of water (1.0 ml) and NH_3_·H_2_O (100 μl, 25%) followed by stirring at room temperature for 15 min. The obtained QAS-MSNs were washed with water for several times. To obtain MSNs, both Ag nanocrystals and QAS molecules in Ag/QAS-MSNs were removed using the above method.

### 2.6 Fourier Transform Infrared Spectroscopy Tests

The samples were collected by centrifugation, dried in the oven overnight at 60°C, and finally used for FTIR testing. FTIR spectra were recorded on a JASCO 6300 spectrophotometer in the solid phase using the KBr pellets technique.

### 2.7 Cellular Experiments

The human HepG2 cells and LO2 cells were maintained in Dulbecco’s modified Eagle medium (DMEM) supplemented with 10% fetal bovine serum, at 37°C, in 5% CO_2_. DMEM and fetal bovine serum were ordered from Hyclone (United States). The entire process of cell culture followed American Type Culture Collection instructions.

### 2.8 Animal Experiments

All animal experiment operations were performed in accordance with the guidelines of the Institutional Animal Care and Use Committee and the care regulations approved by the Administrative Committee of Laboratory Animals of Anhui Medical University (No. LLSC20210778). The tumor model was established by the subcutaneous injection of HepG2 cells (1.0 × 10^5^ cells) into the left axilla of each female Balb/c nude mouse. Tumors were allowed to grow about 50 mm^3^ before use.

### 2.9 *In Vitro* Test of Anticancer Activity

HepG2 cells (1 × 10^4^) were seeded in a 96-well plate for 24 h at 37°C in 5% CO_2_. Then, the cells were treated with nanoparticles at determined concentration. After incubation for another 12 h, cell viabilities were tested using the standard MTT [3-(4,5)-dimethylthiahiazo-2-yl]-2,5-diphenyltetrazolium bromide) assay.

### 2.10 Calcein-AM/Propidium Iodide Staining Assay

HepG2 cells were seeded in a 24-well plate. After 24 h of incubation, Ag/QAS-MSNs (10 and 20 ppm) were added to each well for 12 h. Then, the cells were stained with calcein-AM and propidium iodide (PI) double fluorescent dye, washed three times with PBS, and observed in a fluorescence microscope.

### 2.11 Cell Apoptosis Assay

HepG2 cells were seeded in 12-well plates at a density of 5 × 10^4^ cells/well for 24 h. Ag/QAS-MSNs (10 and 20 ppm) were added to each well. Untreated cells were tested as a negative control. All cells were then washed with PBS and collected by centrifugation. The cells were resuspended in 0.5 ml annexin buffer. Then, all cells were stained with PI and annexin V-FITC containing binding buffer for 15 min and finally detected by flow cytometry.

### 2.12 *In Vivo* Anticancer Therapy

HepG2 cells suspended in PBS (100 μL) injected subcutaneously in the left axilla of female Balb/c mice. When the tumor volume was about 50 mm^3^, the tumor-bearing mice were divided into five randomized groups (*n* = 4 each group), which were PBS treated (control group), Ag/QAS-MSN treated (nanoparticle dose of 20 mg/kg, Ag dose of 0.84 mg/kg, and QAS dose of 2.02 mg/kg), Ag-MSN treated (Ag dose of 0.84 mg/kg), QAS-MSN treated (QAS dose of 2.02 mg/kg), and MSN treated (nanoparticle dose of 20 mg/kg). Within 15 days of treatment, the nude mice were injected with the nanodrugs twice (the aqueous solution of nanodrugs was directly injected into the tumor site every 7 days). The tumor volumes and body weights were tracked for 15 days to estimate the therapeutic performance. The tumor volume was calculated according to the following formula: tumor volume (V) = (tumor length) × (tumor width)^2^/2 (Xu et al., 2022). Furthermore, the major organs of mice (heart, liver, spleen, lung, and kidney) in each group were collected for hematoxylin and eosin (H&E) staining according to the manufacturer’s protocols, followed by examination using a digital microscope.

### 2.13 Test of Antibacterial Activity

The antibacterial activities of the samples were valuated against *E. coli* JM109 and *S. aureus* . These bacteria were cultured in the LB liquid medium at 37°C on a shaker bed until the logarithmic phase [approximately 10^9^ colony forming units (CFU)/ml]. Then, 150 µl of 10^7^ CFU/ml bacterial suspension containing different concentrations of nanoparticles was added in a 96-well microplate and incubated at 37 C with continuous agitation. At given time intervals, the bacterial growth curve was determined by measuring OD at 600 nm. Control experiments were also performed in the presence of empty MCM-41. Meanwhile, 100 µl suspensions of bacteria with different concentrations of nanoparticles were spread on LB agar plates, and then these agar plates were incubated for 14 h at 37°C.

### 2.14 Investigation of the Antibacterial Mechanism by SEM

*S. aureus* (0.5 ml) and *E. coli* bacterial suspensions (0.5 OD) was incubated with Ag/QAS-MSNs (40 ppm) for a given time on a shaking incubator (180 rpm) at 37°C. The bacteria were harvested by centrifugation and washed with water. Then, the bacteria solution was dropped onto a silicon slice and allowed to dry for SEM observation.

### 2.15 Statistical Analysis

Data were described as the mean ± standard deviation, and statistical analysis was performed using one-way analysis and the Bonferroni posttest. Statistical significance was set at *p < 0.05, and significance difference was set at **p < 0.01 and ***p < 0.001.

## 3 Results and Discussion

### 3.1 Synthesis and Characterization of Ag/Quaternary Ammonium Salts Co-Decorated Mesoporous Silica Nanoparticles

Ag/QAS-MSNs were prepared as illustrated in Figure 1A. To effectively encapsulate Ag nanocrystals into the mesostructured silica, TSD was introduced as a chelating agent to capture Ag ions. Then, TEOS and TSD-Ag^+^ complexes were cohydrolyzed to from Ag ions and QAS-MSNs in the presence of QAS, which played the role of a template and surfactant. Further, formalin was added to reduce Ag ions into Ag NPs. The morphology and particle size of Ag/QAS-MSNs were characterized through TEM. The synthesized Ag/QAS-MSNs were spherical particles 30–80 nm in diameter (Figure 1B and Supplementary Figure S1). Lots of small-sized Ag nanoparticles were firmly attached to the surfaces of the MSNs. From the HRTEM images (Figures 1C, D), a uniform and orderly hexagonal array of mesoporous channel ∼2.1 nm in pore diameter, which is the typical mesoporous structure of MCM-41-type MSNs, were observed in Ag/QAS-MSNs, and the diameter of decorated Ag nanoparticles is 2–12 nm. XRD patterns showed that the (111), (200), (220), and (311) planes related to Ag nanoparticles were also found in Ag/QAS-MSNs. In addition, the broad peak at 2θ = 21 could be attributed to the amorphous porous silica (Aznar et al., 2009). These results preliminary prove that Ag/QAS-MSNs were successfully prepared using our one-pot method. Moreover, the porosity of MSNs was also investigated by N_2_ adsorption/desorption measurements. From Supplementary Figure S2, the Brunauer–Emmett–Teller (BET) surface area, pore volume, and pore diameter of the MSNs were 715.5 m^2^/g, 0.64 m^3^/g, and 2.2 nm, respectively.

**FIGURE 2:**
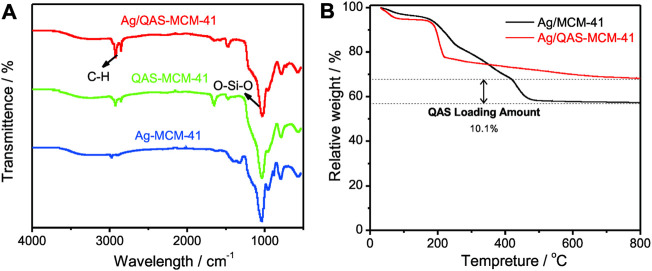
**(A)** FIIR spectra of Ag/QAS-MSNs, QAS-MSNs, and Ag-MSNs. **(B)** Thermogravimetric analysis (TGA) curves of Ag/QAS-MSNs and MSNs.

Figure 2 shows the FTIR spectra of Ag/QAS-MSNs, QAS-MSNs, and Ag-MSNs. The characteristic peak at 1,090 cm^−1^ was assigned to Si–O–Si stretching vibration of MSNs (Fang et al., 2012), and the characteristic peaks at 2,930 and 2,850 cm^−1^ were assigned to C–H stretching vibration coming from QAS and TSD (Fang et al., 2016). All the characteristic adsorption bands were observed in the FTIR spectra of these samples. However, the intensity of the peaks concerning the C–H band in Ag/QAS-MSNs was dramatically higher than that in Ag-MSNs, indicating that the QAS molecules were successfully encapsulated into the mesostructured silica. Thermogravimetric analysis (TGA) was further used for quantitative analysis of the amounts of QAS in Ag/QAS-MSNs. From Figure 2B, it was obvious that Ag/QAS-MSNs have two weight loss stages when heated in air. A weight loss below 400°C might be due to the release of high–boiling point solvent absorbed on the nanoparticles’ surface, while a further weight loss when temperature is up to 400°C probably corresponded to the burning of the QAS. Based on the TGA curves of Ag/QAS-MSNs and Ag-MSNs, the encapsulation of QAS in Ag/QAS-MSNs was calculated as 10.1 wt%. Additionally, the loading amount of Ag in Ag/QAS-MSNs determined by ICP-MS was ∼4.2 wt% (Supplementary Table S1).

### 3.2 *In Vitro* Anticancer Activity of Ag/Quaternary Ammonium Salts Co-Decorated MSNs

**FIGURE 3:**
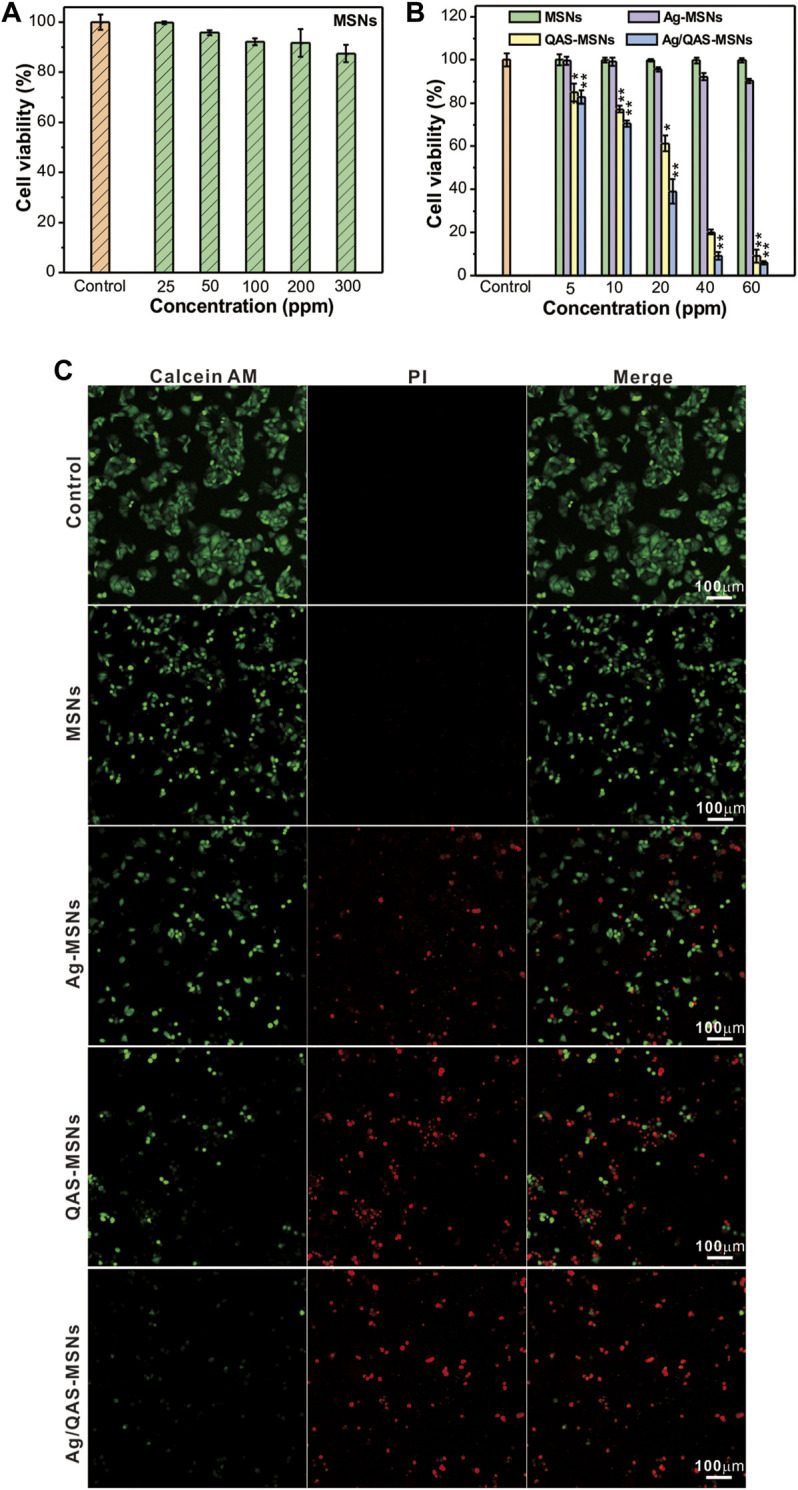
**(A,B)** Relative survival of HepG2 cells treated with different concentrations of MSNs, Ag-MSNs, QAS-MSNs, and Ag/QAS-MSNs for 24 h. **(C)** Fluorescent images of HepG2 cells incubated with MSNs, Ag-MSNs, QAS-MSNs, and Ag/QAS-MSNs at concentration of 40 ppm. The cells were stained with calcein-AM and PI double fluorescent dye. Green indicates live cells, and red indicates dead cells. All data are presented as the mean of three different measurements ± SD, asterisks (*) represent the significant difference from the control group using the Bonferroni posttest (*p* ≤ 0.05) and asterisks (**) *p* < 0.01.

In order to investigate the cytotoxicity of the MSN vehicles *in vitro*, HepG2 cells and LO2 cells (normal human live cells) were incubated with different concentrations of MSNs and determined using a standard MTT assay (Figure 3 and Supplementary Figure S2). As shown in Figure 3A, no obvious cytotoxicity was detected for the MSN vehicles without QAS and Ag NP loading even after 24-h incubation at the concentration of 300 ppm, which suggested the biocompatibility of the MCM-41-type vehicles. Results from Figure 3B showed that the cell viabilities exhibited a marked decline in a dose-dependent manner in the presence of Ag/QAS-MSNs, QAS-MSNs, and Ag-MSNs, and Ag/QAS-MSNs showed higher antitumor effect than QAS-MSNs and Ag-MSNs at the same condition. For instance, the viability of cells treated with Ag/QAS-MSNs at the concentration of 40 ppm [QAS: 4.04 ppm (10.1 wt%) silver: 1.68 ppm (4.2 wt%)] was decreased to 9.1%, while that of cells treated with QAS-MSNs (QAS: 4.04 ppm) and Ag-MSNs (Ag: 1.68 ppm) were only 20.2 and 92.2%, respectively. It was intuitively observed, using the calcein-AM/PI staining double staining method, that cells incubated with MSNs mainly displayed green fluorescence (color in green indicates live cells, and color in red indicates dead cells). The Ag-MSNs group showed a small amount of red fluorescent cells, while both the QAS-MSNs and Ag/QAS-MSNs groups exhibited almost dead cells, which was consistent with the data of CCK-8 assay.

**FIGURE 4:**
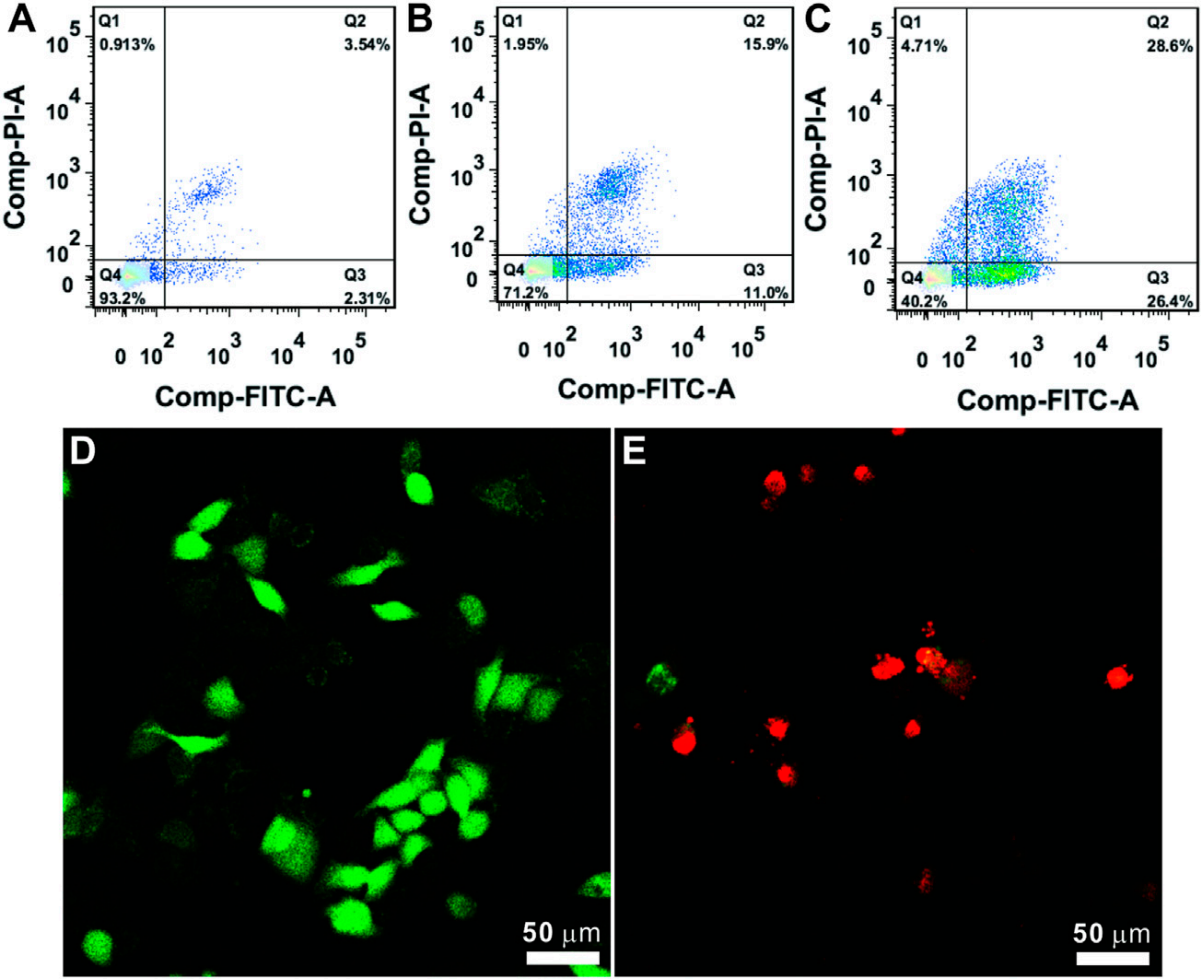
**(A–C)** Cell apoptosis analysis using the PI/annexin V-FITC double staining method: **(A)** control; **(B)** Ag/QAS-MSNs, 10 ppm; **(C)** Ag/QAS-MSNs, 20 ppm. Q1: necrosis, Q2: late apoptosis, Q3: early apoptosis, Q4: live. **(D,E)** Cell morphology of the dying cells induced by Ag/QAS-MSNs suggested a form of apoptosis: **(D)** control; **(E)** Ag/QAS-MSNs, 20 ppm.

Further, the possible Ag/QAS-MSN-induced cell death mechanism was investigated by the flow cytometry analysis. Results shown in Figure 4C indicated that cell populations in early and late apoptosis were increased dose-dependently in the presence of Ag/QAS-MSNs. As seen, the percentage in apoptosis for the Ag/QAS-MSN-treated cells increased from 26.9% (early and late apoptosis) at 10 ppm to 55% at 20 ppm. From the fluorescence images, the green fluorescence indicates no morphological changes (control cells). The cells treated with Ag/QAS-MSNs exhibited significant changes including cell shrinkage, cell membrane blebbing, DNA fragmentation of apoptotic bodies. These results demonstrate that cell death induced by Ag/QAS-MSNs is mainly caused by apoptosis.

### 3.3 *In Vivo* Anticancer Activity and Biosafety of Ag/Quaternary Ammonium Salts Co-Decorated Mesoporous Silica Nanoparticles

**FIGURE 5:**
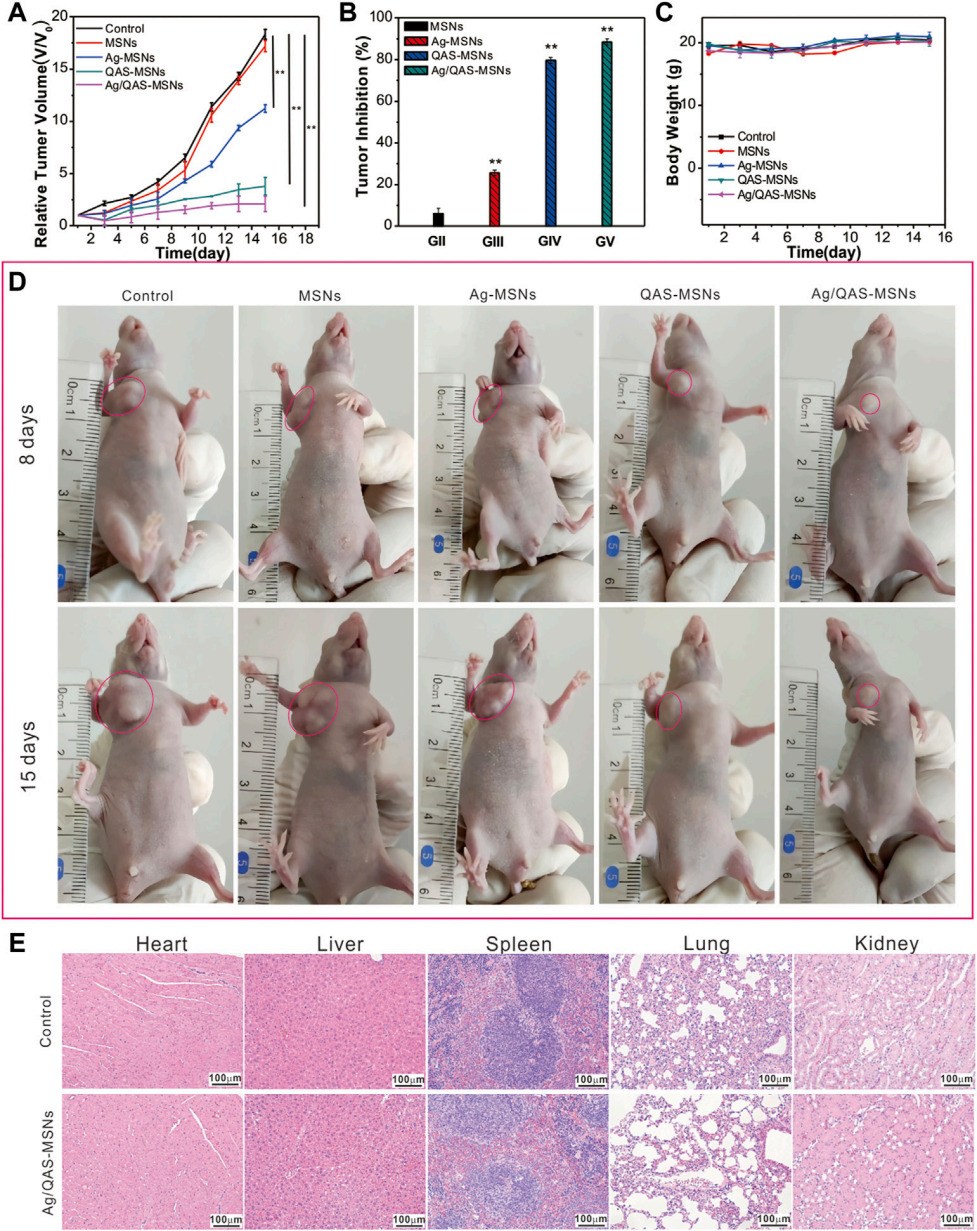
The tumor growth curves **(A)**, the relative tumor volume **(B)**, and the body weights **(C)** of mice after the treatment with PBS as control, MSNs, Ag-MSNs, QAS-MSNs, and Ag/QAS-MSNs. **(D)** Representative photos of the tumor-bearing mice treated by five treatment groups after 8 and 15 days (the red circles point to the tumor site). **(E)** H&E staining images of major organs after treatment with PBS and Ag/QAS-MSNs. All data are presented as the mean of three different measurements ±SD, asterisks (*) represent the significant difference from the control group using the Bonferroni posttest (*p* ≤ 0.05) and asterisks (**) *p* < 0.01.

The HepG2 tumor-bearing mice are randomly separated into five groups, namely, PBS, MSNs, Ag-MSNs, QAS-MSNs, and Ag/QAS-MSNs. As illustrated in Figure 5A, the relative tumor sizes decreased considerably upon treatment with Ag/QAS-MSNs compared with those upon treatment with PBS, MSNs, Ag-MSNs, and QAS-MSNs. About 88.5% of tumor inhibition ratio was shown by the treatment with Ag/QAS-MSNs for 15 days (Figure 5B), whereas Ag-MSNs and QAS-MSNs treatments achieved only 25.7 and 79.7% of tumor inhibition ratio, respectively. In agreement with these results, the representative images of tumor-bearing mice of each group are shown in Figure 5D. During 15-day treatments, all groups did not show significant body weight changes. H&E staining of the major organs treated with PBS (control group), MSNs, Ag-MSNs, QAS-MSNs, and Ag/QAS-MSNs exhibited no histopathological abnormalities, which indicated that the above nanomaterials nearly had no side effects on the health of the mice (Figure 5E and Supplementary Figure S4). Therefore, these results revealed that Ag/QAS-MSNs can be considered a promising anticancer agent in tumor treatment without producing toxicity.

### 3.4 *In Vitro* Antibacterial Activity and Its Mechanism

**FIGURE 6:**
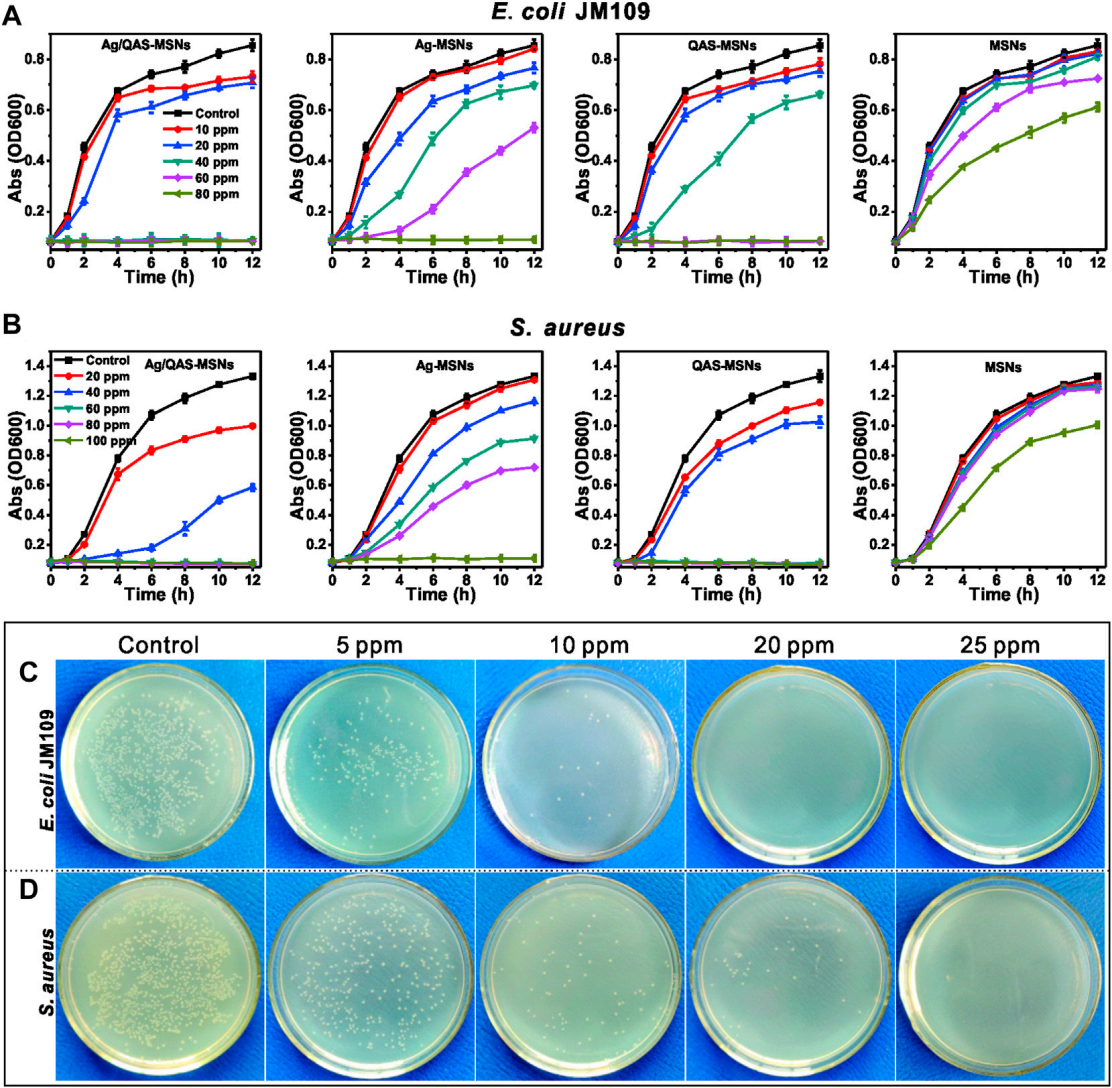
Growth curves of *E. coli* **(A)** and *S. aureus* **(B)** in LB liquid medium in the presence of different concentrations of MSNs, Ag-MSNs, QAS-MSNs, and Ag/QAS-MSNs. *E. coli* **(C)** and *S. aureus* **(D)** grown on agar plates at different concentrations of Ag/QAS-MSNs.

The antibacterial activity of the nanomaterials was evaluated against selected pathogenic Gram-negative (*E. coli* JM109) and Gram-positive strains ( *S. aureus* ) in both liquid media and LB agar plates. The bacterial strains were incubated with MSNs, Ag-MSNs, bare Ag NPs, pure QAS, QAS-MSNs and Ag/QAS-MSNs at the same equivalent concentrations. The results demonstrated that Ag/QAS-MSNs displayed a strongest antibacterial activity when compared with Ag-MSNs, QAS-MSNs, bare Ag NPs, and pure QAS (Figure 6 and Supplementary Figure S5). As can be seen from Figure 6A, the proliferation of *E. coli* was completely inhibited in the presence Ag/QAS-MSNs at the concentration of 40 ppm, whereas no significant antibacterial activity was observed in the groups of MSNs, Ag-MSNs, and QAS-MSNs. In the case of *S. aureus* (Figure 6B), 60 ppm of Ag/QAS-MSNs was needed to completely suppress bacterial growth in liquid media, which suggested that the Gram-positive *S. aureus* was more tolerant than the Gram-negative *E. coli* to Ag/QAS-MSNs. This resistance may be due to the thicker cell wall and different chemical components of Gram-positive bacteria (Buckley et al., 2010; Fang et al., 2014). Additionally, QAS-MSNs appeared to have a slightly higher antibacterial effect against the two pathogenic bacteria than did Ag-MSNs at all test concentrations. Figures 6C, D depict the formation of bacterial colonies on the control and Ag/QAS-MSNs after 14-h incubation. It was obviously seen that Ag/QAS-MSNs can effectively inhibit bacterial growth in a concentration-dependent manner. The growth of bacterial colonies was completely prevented, when the concentration of Ag/QAS-MSNs was increased to 20 ppm for *E. coli* and 25 ppm for *S. aureus* , respectively. Hence, the prepared Ag/QAS-MSNs can act as an effective antibacterial agent.

**FIGURE 7:**
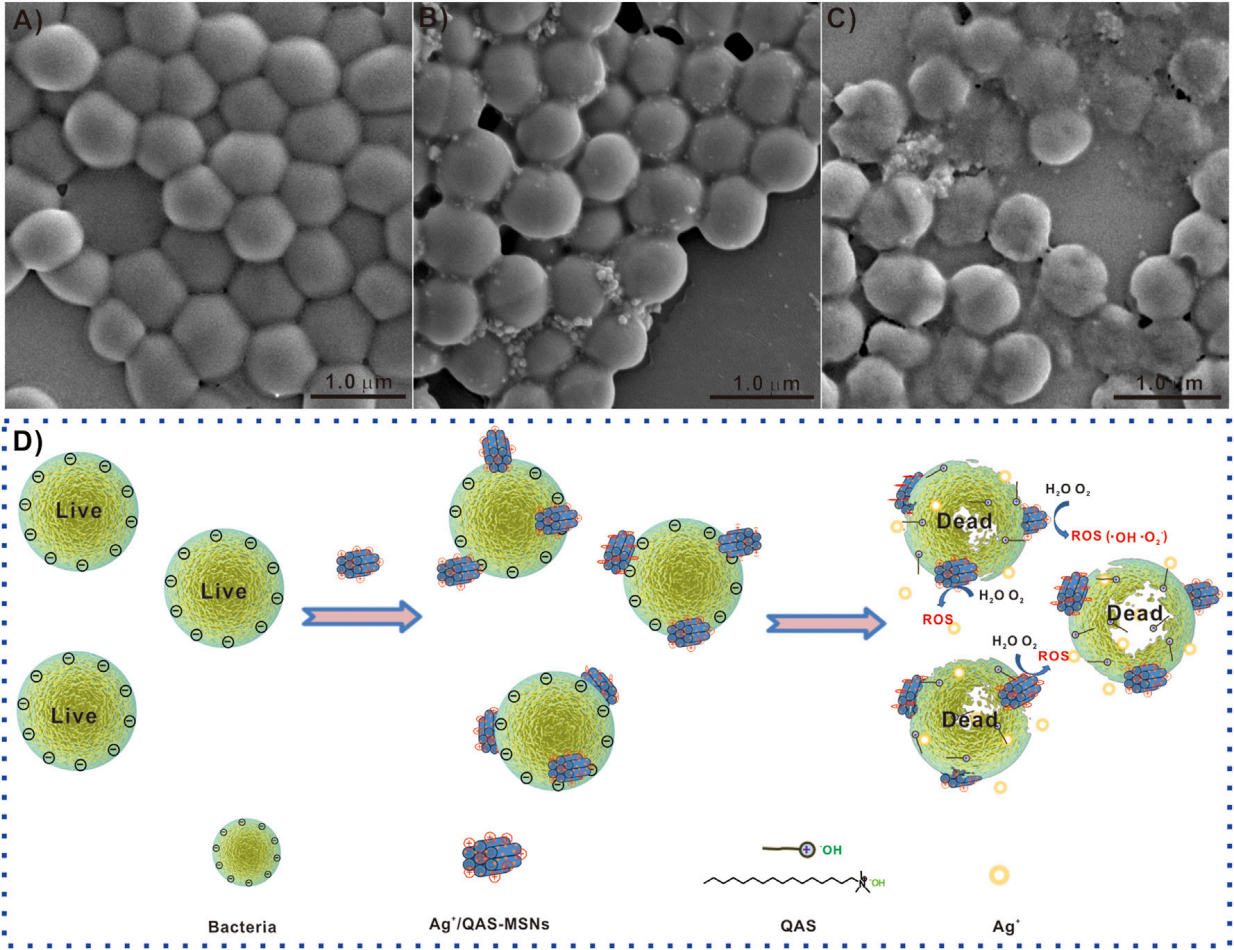
SEM images of *S. aureus* treated with Ag/QAS-MSNs: **(A)** 0 ppm (control), **(B)** 40 ppm for 30 min, and **(C)** 40 ppm for 8 h. **(D)** The possible antibacterial mechanism of Ag/QAs-MSNs.

To further investigate the antibacterial mechanism of Ag/QAS-MSNs, the mixture of *S. aureus* and nanomaterials was used for SEM analysis at different incubation times. After incubation for 30 min, SEM images (Figure 7B and Supplementary Figure S7) reveal that *S. aureus* still maintained intact cell wall structure, and lots of Ag/QAS-MSNs adhered to the outer membrane of the cells. This was because the negatively charged bacterial cell wall can easily adhere to materials carrying positive charges (Supplementary Figure S8) (Fang et al., 2017; Hu et al., 2020). However, with the extension of the incubation times (Figure 7C), destruction and degradation of the cell membrane were observed, and most bacterial cells had no intact cell morphology indicating dead cells. These phenomena propose that the possible antibacterial mechanisms are as follows (Figure 7D): First, Ag/QAS-MSNs with positive charges adhere to the negatively charged bacterial cell wall via electrostatic interaction and cause injury to the cytomembrane (Hayden et al., 2012; Chen et al., 2014). Subsequently, the sustainable release of Ag ions and QAS from Ag/QAS-MSNs can directly lead to membrane damage and cell death. However, the Ag ions may also generate reactive oxygen species inducing membrane lipid peroxidation, DNA damage, and protein denaturation (Eckhardt et al., 2013).

## 4 Conclusion

In summary, we describe the synthesis of Ag/QAS-MSNs *via* the one-pot co-condensation method for anti-bacteria and *in vivo* tumor treatment. Our experimental results demonstrated that Ag/QAS-MSNs possess excellent antibacterial activity and anticancer property. The excellent anticancer and antibacterial activity of Ag/QAS-MSNs could be ascribed to the synergistic effect of Ag ions and QAS. Hence, we state that Ag/QAS-MSNs can emerge as promising agents for effective treatment of tumors and the related bacterial infections.

## Data Availability

The original contributions presented in the study are included in the article/Supplementary Material, and further inquiries can be directed to the corresponding authors.
