# Supplementary material for: One-Pot Synthesis of Ag/Quaternary Ammonium Salt Co-Decorated Mesoporous Silica Nanoparticles for Synergistic Treatment of Cancer and Bacterial Infections
Source: Front Bioeng Biotechnol. 2022 Jul 19;10:875317. doi: 10.3389/fbioe.2022.875317 (PMC9344533; doi:10.3389/fbioe.2022.875317)
Supplement: Supplementary file 1 [file DataSheet1.docx]

Supporting Information

## One-pot synthesis of Ag/QAS co-decorated mesoporous silica nanoparticles for synergistic treatment of cancer and bacterial infections

Hanyuan Zhang ^a #^ , Jianxiang Xu ^b #^, Xu Zhang ^b^, Teng Wang ^b^, Dairan Zhou ^b^, Wei Shu ^b^, Tingting Zhao ^b *^, Weijun Fang ^b *^

^a^ Department of Orthopedics, Department of Sports Medicine and Arthroscopic Surgery, The First Affiliated Hospital of Anhui Medical University, Hefei 230022, China

^b^ School of Basic Medical Sciences, Anhui Medical University, Hefei 230032, Anhui, China

^#^ These authors contributed equally to this work.

Corresponding Author

* (Tingting Zhao) E -mail: [ttzhao@ahmu.edu.cn](mailto:ttzhao@ahmu.edu.cn)

*(Weijun Fang) E-mail: [fangweijun@ahmu.edu.cn](mailto:fangweijun@ahmu.edu.cn); [wjfang81@163.com](mailto:wjfang81@163.com)

Phone: +86551 65161138


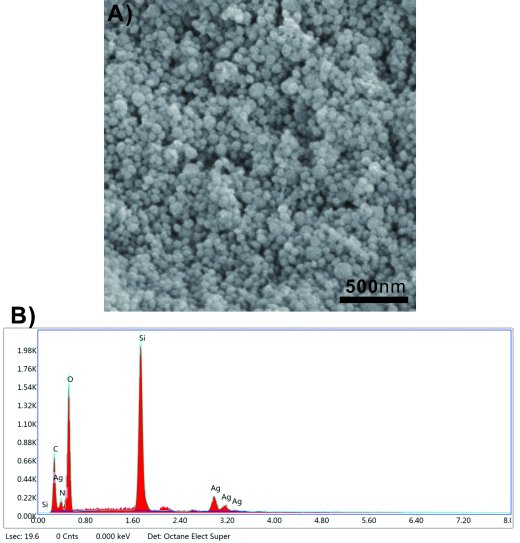


**Figure S1.** A) SEM image of Ag/QAS-MSNs; B) EDX analysis of Ag/QAS-MSNs: EDX spectrum revealed the presence of Ag, Si, N, C and O which confirm the formation of Ag/QAS-MSNs.

**
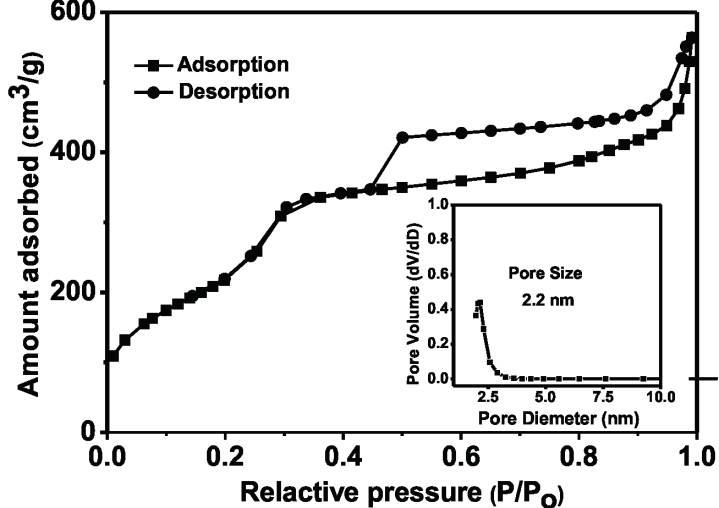
**

**Figure S2.** N_2_ adsorption/desorption isotherm and the pore size distribution (inset) of [Na]-HMAS


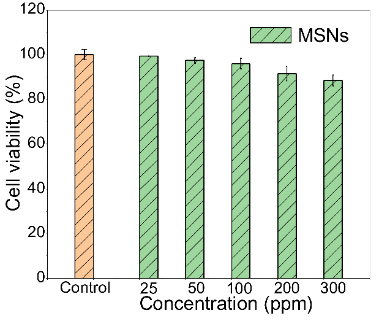


Figure S3. Relative survival of LO2 cells treated with different concentrations of MSNs for 24 h.


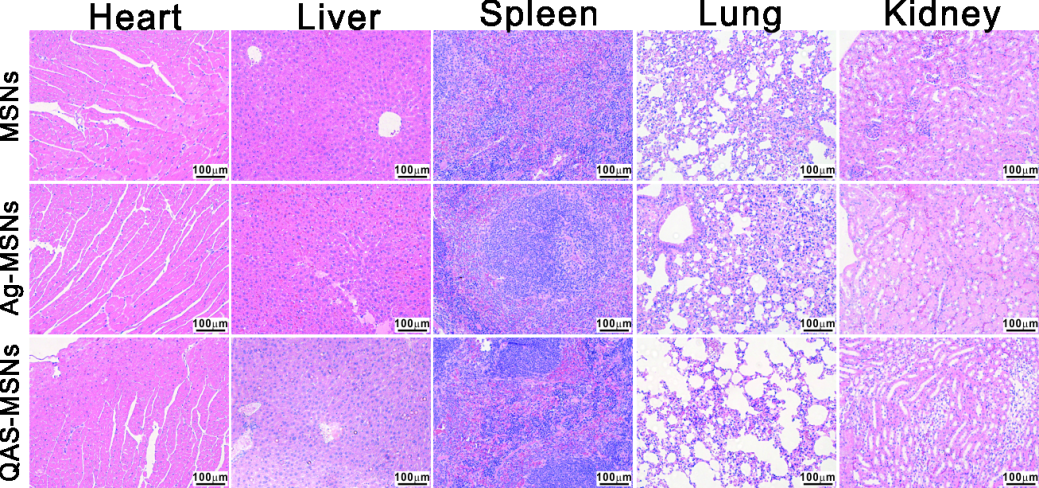


Figure S4. H&E staining images of major organs after treatment with MSNs, Ag-MSNs and QAS-MSNs.


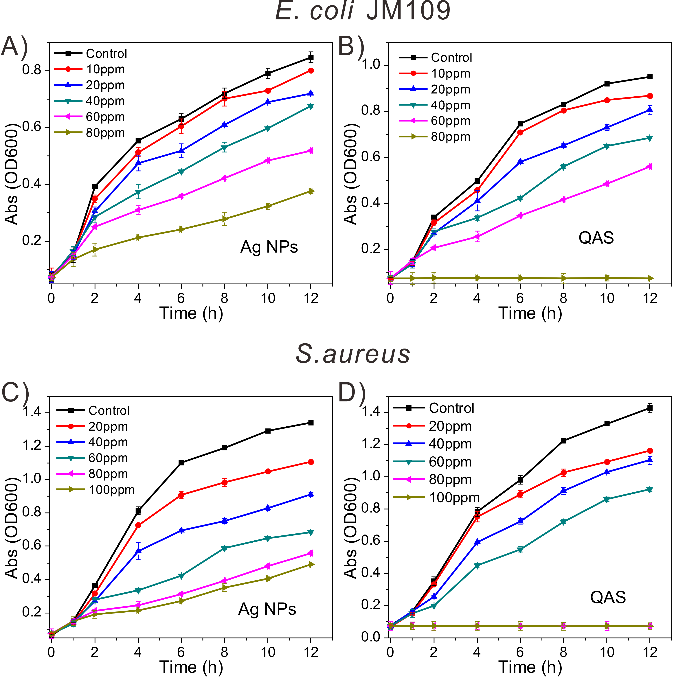


Figure S5. Growth curves of E. coli JM109 (A-B) and S. aureus (C-D) in LB liquid medium in the presence of bare Ag NPs (A, C) and pure QAS (B, D) at the equivalent concentrations.

**
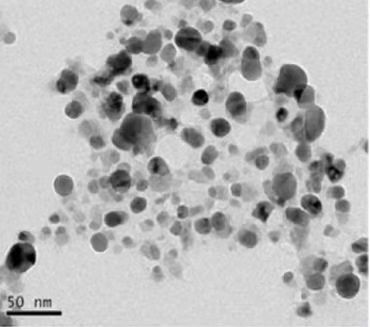
**

**Figure S6.** TEM image of bare Ag NPs.


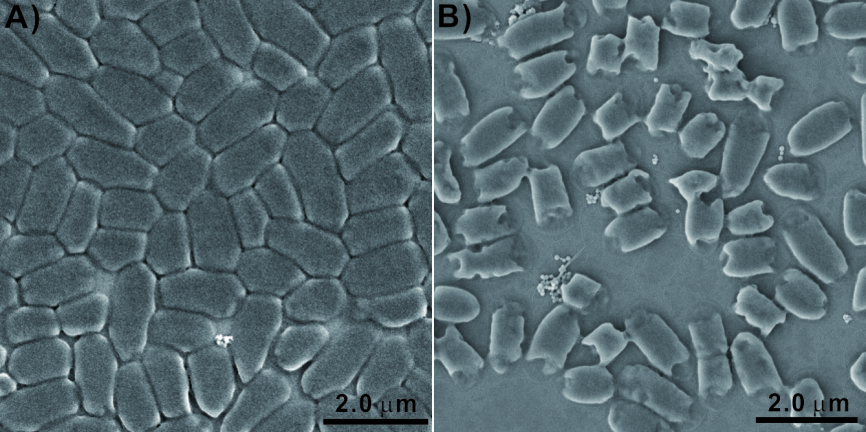


**Figure S7.** SEM images of *E. coli JM109* treated with Ag/QAS-MSNs: A) 0 ppm (control) and B) 40 ppm for 30 min.


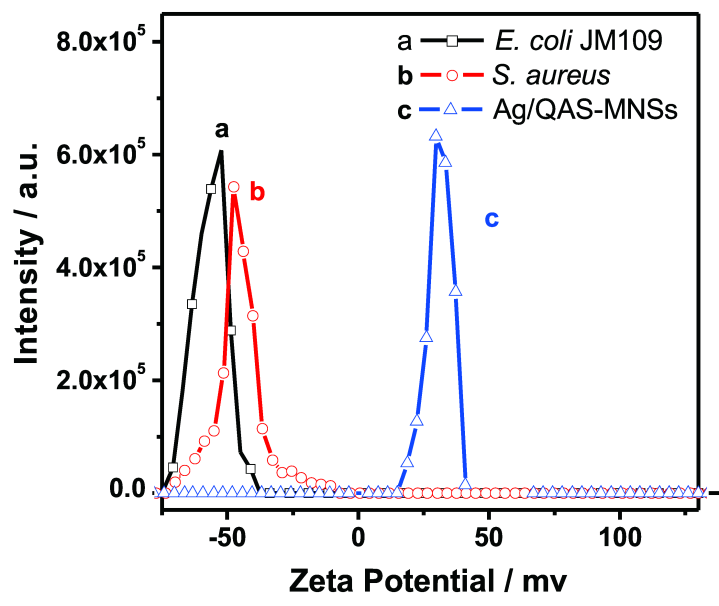


**Figure S8.** Zeta potential of *E. coli* JM109 (-52.3.1 mV), [*S. aureus*](http://www.youdao.com/w/staphylococcus%20aureus/#keyfrom=E2Ctranslation) (-47.6 mV) and Ag/QAS-MSNs (+29.8 mV).

**Table S1.** The total silver content in Ag/QAS-MSNs determined by ICP-MS.

|  | **Total Nanomaterials( mg)** | **Total**  **Silver (mg)** | **Silver Loading Amount(wt%)** |
| --- | --- | --- | --- |
| **Ag/QAS-MSNs** | ~ 10 | ~ 0.416 | ~ 4.2 |
